# The diagnosis and phacoemulsification in combination with intraocular lens implantation for an Axenfeld–Rieger syndrome patient with small cornea: a case report

**DOI:** 10.1186/s12886-020-01406-z

**Published:** 2020-04-15

**Authors:** Yajuan Ma, Xingdi Wu, Shuang Ni, Xiang Chen, Suhong He, Wen Xu

**Affiliations:** 1 Eye Center, the Second Affiliated Hospital, School of Medicine, Zhejiang University, Hangzhou, 310009 China; 2 Zhejiang Rongjun Hospital, Jiaxing, 314000 China; 3 Suichang Hospital of Traditional Chinese Medicine, Suichang, 323300 China

**Keywords:** Axenfeld–Rieger syndrome, Cataract, Small cornea, Phacoemulsification, Autosomal dominant

## Abstract

**Background:**

Axenfeld-Rieger syndrome (ARS) is a congenital disease with a series of developmental abnormalities, and no case of ARS with cataract and small cornea has been reported in previous studies. In the present report, we aimed to describe the diagnosis and phacoemulsification of an ARS patient with small cornea.

**Case presentation:**

A 58-year-old Han Chinese male patient who was referred to Eye Center of the Second Affiliated Hospital of Zhejiang University Medical College was diagnosed with ARS. Systemic and ophthalmic examination and genetic testing were performed. The slit-lamp microscopic examination of anterior segment showed obvious nuclear cataract, iris lesions, and the abnormal cornea of both eyes with small transversal and longitudinal diameters. ARS with bilateral complicated cataract and small cornea was diagnosed. Microincision-phacoemulsification in combination with intraocular lens implantation was performed on his left eye. After successful surgery of his left eye, the best-corrected visual acuity (BCVA) was obviously improved from 2 to 0.5 (LogMAR). A transient elevation of intraocular pressure (IOP) was controlled with medication.

**Conclusions:**

Through genetic testing, a known pathogenic mutation NM_153427.2:c.272G > A was detected on the PITX2 gene; and an unknown mutation NM_001453.2:c.1063C > T was detected on FOXC1 gene. For the ARS patient with complicated cataract, the visual acuity was increased by phacoemulsificasion in combination with microincision.

## Background

Axenfeld-Rieger syndrome (ARS) is a congenital disease with a series of developmental abnormalities, including anterior segment dysgenesis and abnormalities in the extraocular tissues, from which the neural crest cells originate [1]. Such disorder is transmitted in an autosomal-dominant manner, with an estimated prevalence of 1 in 50,000 to 100,000 newborns [2]. Ocular features of ARS include featured posterior embryotoxon, changes in iris, cornea and the anterior angle. Developmental glaucoma is often caused by the presence of posterior embryotoxon and abnormal anterior angle development, which occurs in nearly 50% of the cases [3]. Systemic findings include mild midface abnormalities, dental and cardiovascular abnormalities, and redundant umbilical skin [4]. Till now, only very few cases have reported the clinical features of ARS, with retinal detachment, keratoconus, developmental glaucoma and so on [3, 5–8]. In the present report, small cornea, iris lesions, pupil deformation and cataract were first discovered after the slit microscope examination, and through careful examination of the atrial Angle, the posterior corneal embryonic ring was also found. Combined with the features of the eyes, the systemic characteristics were also obtained: dental abnormalities of hypodontia in the upper, lower teeth, and redundant periumbilical skin. The patient was diagnosed as ARS based on the characteristics of the eyes and exophthalmic system, and then genetic testing was used to verify and obtain the genetic mutation information of the case. We aimed to describe the diagnosis and surgical treatment of an ARS patient with cataract who had a pair of small corneas.

## Case presentation

A 58-year-old Han Chinese male patient was referred to Eye Center of the Second Affiliated Hospital of Zhejiang University Medical College on February 21st, 2019. His bilateral vision was progressively decreased for more than 5 years. He received bilateral trabeculectomy 30 years ago and did not receive any anti-glaucoma medications from then on. The patient was first admitted to a local hospital 30 years ago due to “blurred vision”. He was found to have small cornea, multiple pupil disease, iris atrophy and high intraocular pressure. According to clinical ophthalmic features, he was diagnosed as Rieger syndrome and received anti-glaucoma surgery. Half a year ago, he was recommended to Dr. Xu for “gradual aggravation of blurred vision” and diagnosed as ARS based on the medical history, ocular and systemic clinical features.

His parents died when he was young. His sister had glaucoma on both eyes and received surgical treatment. His brother was blind because of boxing injury when he was young. His daughter and son were normal without ARS phenotype. The present study was adhered to the tenets of the Declaration of Helsinki. An informed consent was obtained from the patient.

**Fig. 1:**
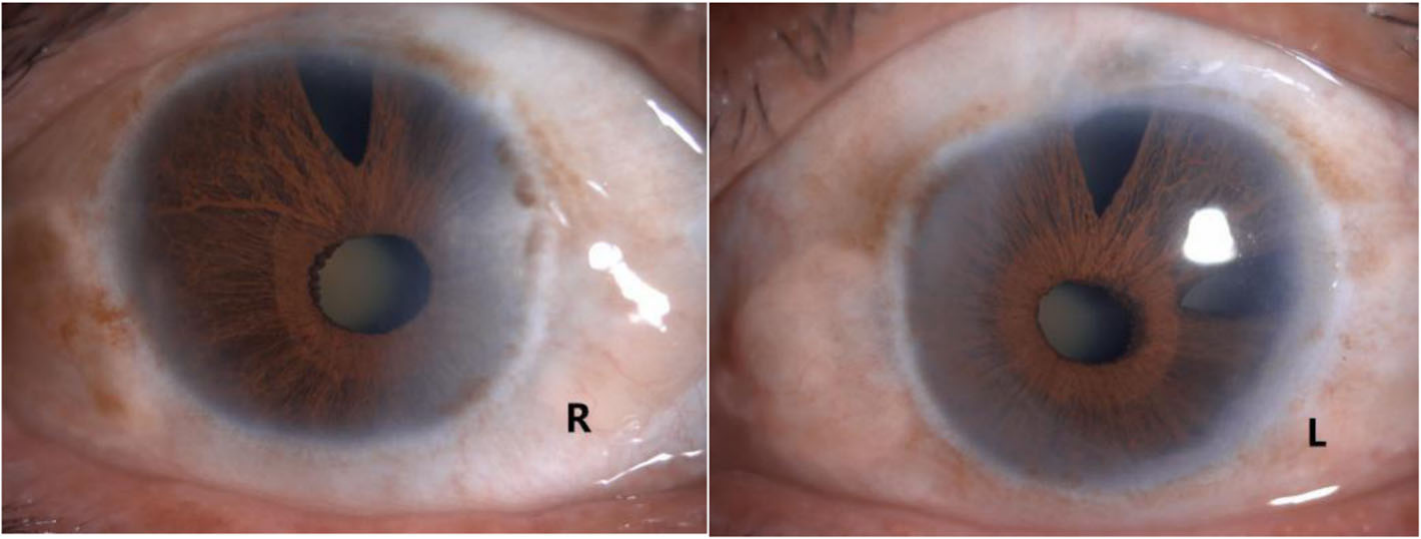
Obvious nuclear cataract, iris lesions, and the abnormal cornea of both eyes which involved to PMD and morphological irregularity were shown via the slit-lamp microscope examination of anterior segment. The iris changes included the atrophy and loose arrangement of the iris, the displaced pupil beneath the nose and hiatus formation

**Fig. 2:**
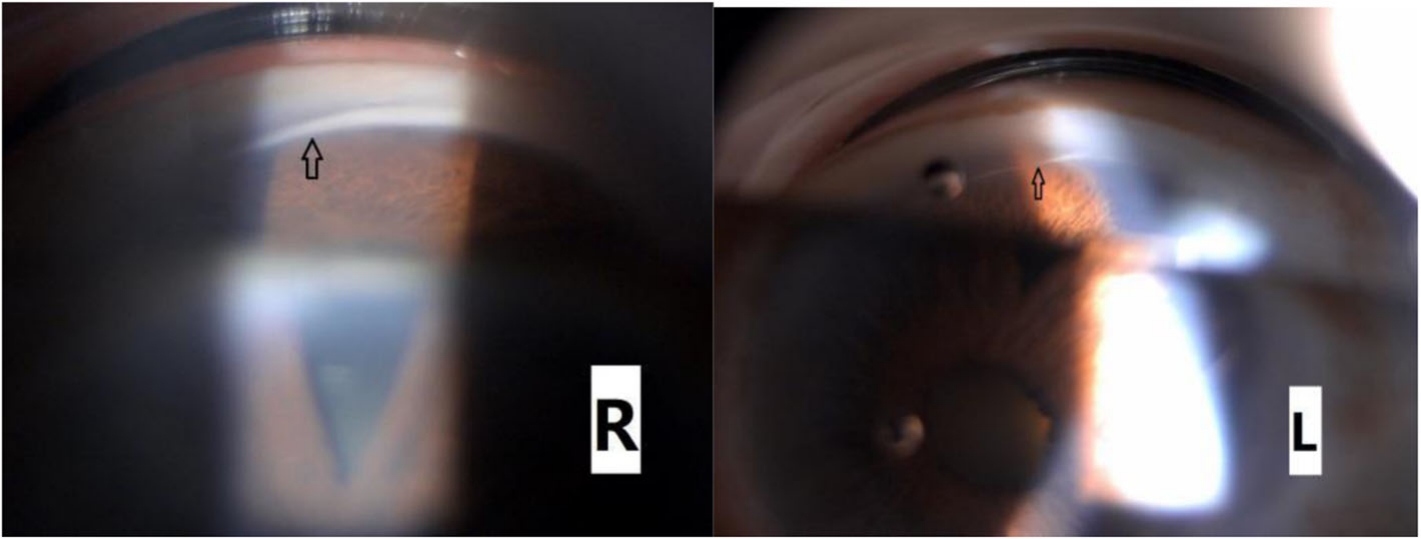
Gonioscopy suggested characteristic corneal posterior embryotoxon

**Fig. 3:**
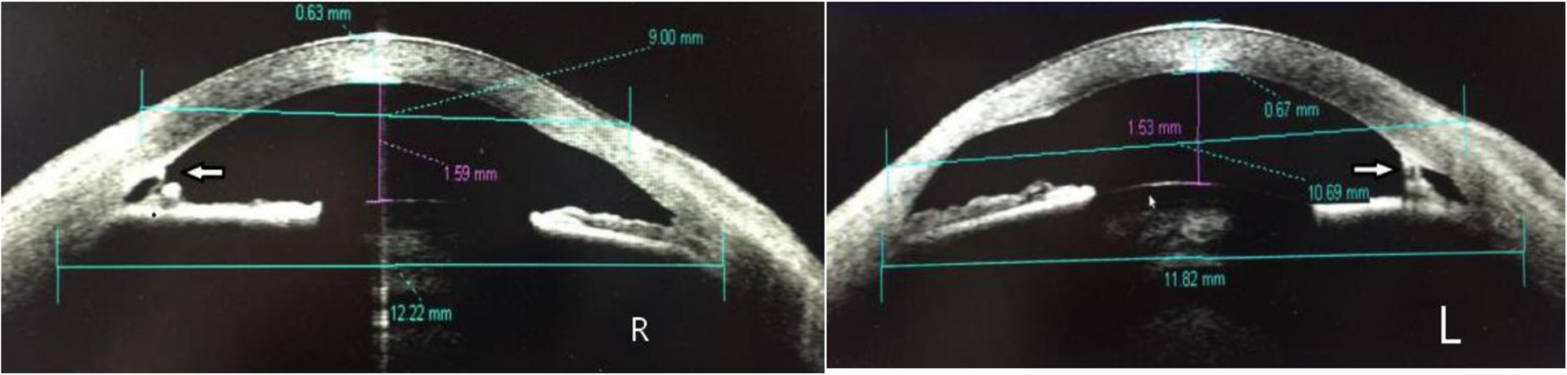
Anterior segment imaging examination showed adhesion of the cornea to the iris in both eyes (white arrow)

**Fig. 4:**
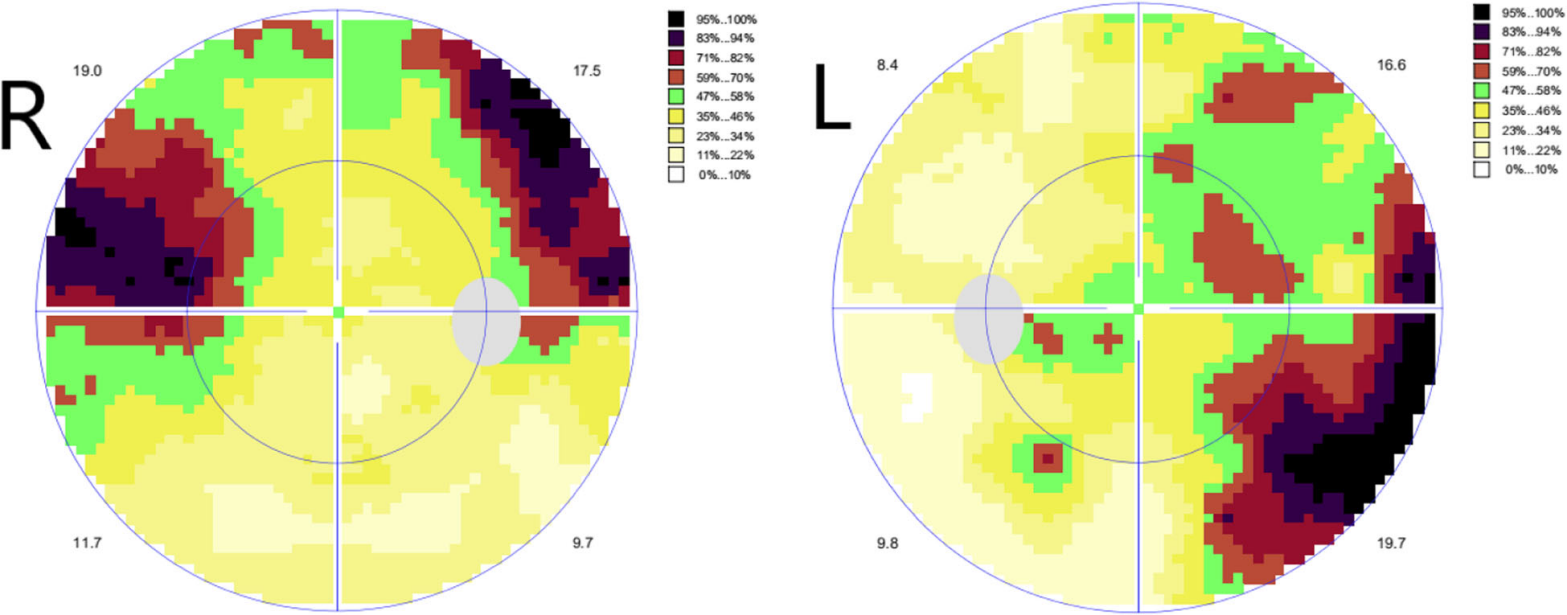
The central visual field examination revealed a defect in the visual field of both eyes

A comprehensive ophthalmic examination was performed (Table 1). The best-corrected visual acuity (BCVA) was 2.0 (logMAR) bilaterally, while the intraocular pressure (IOP) was 17.0 mmHg in the right eye and 16.5 mmHg in the left eye (Goldmann Applanation Tonometry, Suzhou City, Jiangsu Province, China). The slit-lamp microscopy of anterior segment showed obvious nuclear cataract (C_2_N_3_P_1_ with LOCSII), iris lesions, and the abnormal cornea of both eyes which involved in pellucid marginal degeneration (PMD) and morphological irregularity (Fig. 1). Gonioscopy suggested characteristic corneal posterior embryotoxon (Fig. 2). Anterior segment imaging examination (Visante OCT, Zeiss, Germany) showed adhesion of the cornea to the iris in both eyes (Fig. 3). The iris changes included iris atrophy, loose arrangement and the nasal-displaced pupil with hiatus formation (Fig. 1). The value of flat corneal curvature (K) was 36.8 D in the right eye and 38.2 D in the left eye through anterior segment analysis diagnostic system (Pentacam, Oculus, Germany). An ultrasonic measurement (Quantel, Sinescan, France) showed that the axial length (AXL) was 28.18 mm in the right eye and 29.26 mm in the left eye. The central visual field examination revealed a defect in the visual field of both eyes (Fig. 4).

**Table 1:** Biometric parameters revealed by detailed opthalmological examination

Parameters	Right eye	Left eye
Flat corneal curvature K(D)	36.8	38.2
Axial length (mm)	28.18	29.26
Horizontal corneal diameter (mm)	8.9	8.8
Vertical corneal diameter (mm)	8.0	7.8
Anterior chamber (mm)	1.84	1.53
Posterior embryotoxon	Yes	Yes
Cup/disc	0.7	0.7
Central visual field defect	Yes	Yes

**Fig. 5:**
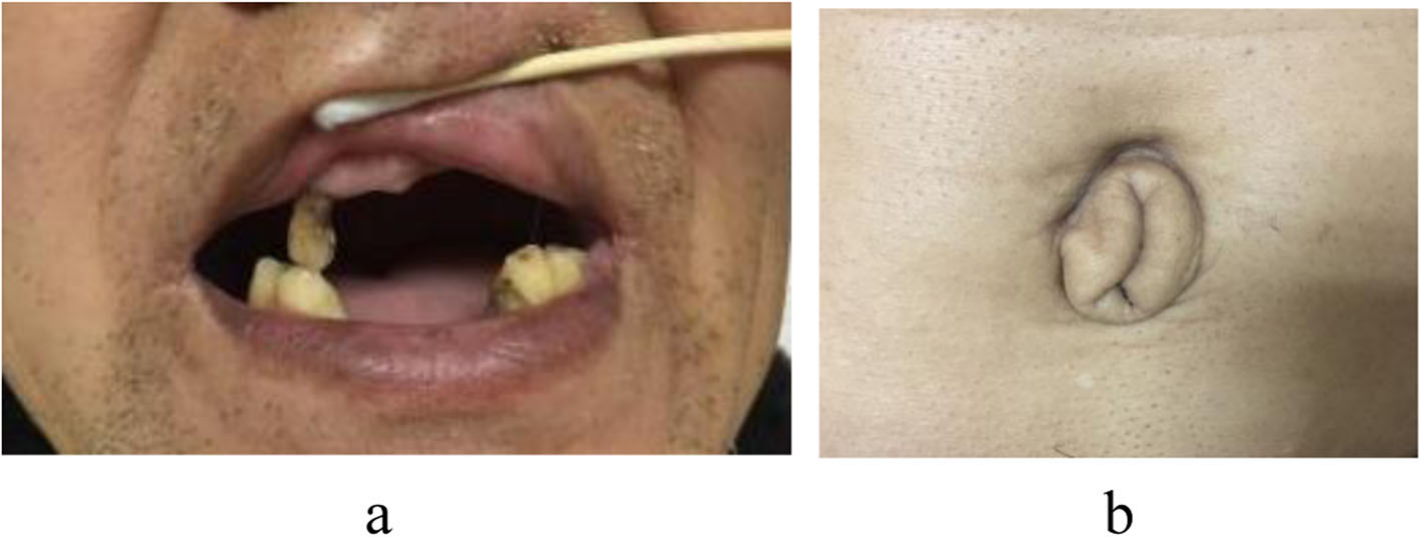
**a** Dental abnormalities of hypodontia of the upper and lower teeth in permanent dentition. **b** Redundant periumbilical skin

The patient had dental abnormalities of hypodontia in the upper and lower teeth in permanent dentition (Fig. 5a). In addition, the patient presented with redundant periumbilical skin (Fig. 5b).

About 2 mL of peripheral blood samples was collected from this patient into Vacutainer tubes (Becton-Dickinson, Franklin Lakes, NJ) containing EDTA and sent to BGI Genomics (BGI-Shenzhen, Shenzhen 518,083, China) for clinical exome detection. Through genetic testing, a known pathogenic mutation NM_153427.2:c.272G > A was detected on the PITX2 gene, and an unknown mutation NM_001453.2: c.1063C > T was detected on FOXC1 gene.

**Fig. 6:**
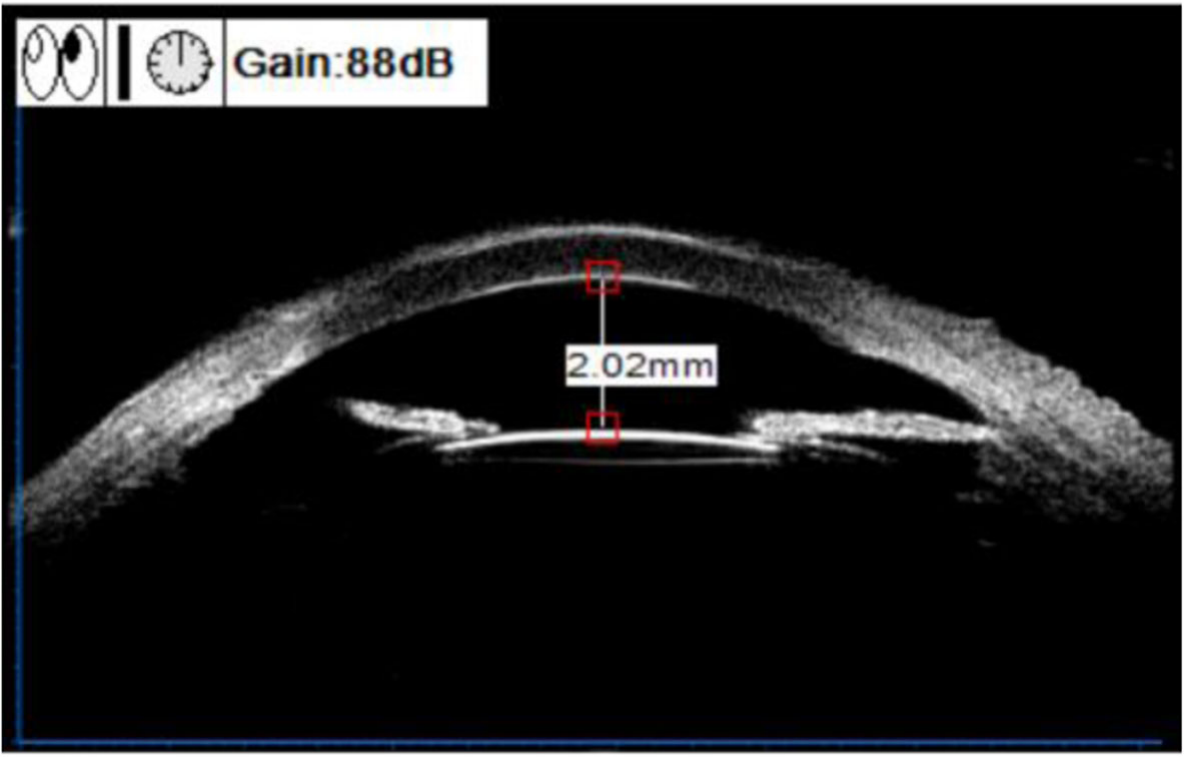
At 1 week after surgery, UBM examination showed that the intraocular lens (IOL) was located in the capsular bag, with a anterior chamber depth of 2.02 mm, which was significantly deeper than the preoperative depth of 1.59 mm, and the iris hole was visible

The patient received a microincision-phacoemulsification (Millenium, Bausch & Lomb) and intraocular lens implantation for the cataract on his left eye in Eye Center, the Second Affiliated Hospital of Zhejiang University on March 7th, 2019. The operation was performed under topical anesthesia. A self-sealing temporal limbal micro-incision (2.0 mm), capsulorhexis of 5.0 mm in diameter, and the phacoemulsification using the stop-and-chop technique, as well as an implantation of a hydrophobic aspheric posterior-chamber intraocular lens with + 14.0 Diopter (Akreos AO, Bausch&Lomb MI60,USA), were performed. Data regarding the microscope-light exposure time (27 min), the average phacoemulsification time (APT) (1.58 min) and the intraoperative measurements of phaco energy (MPE) (10%) were recorded at the end of the surgery. Postoperative treatment included levofloxacin eye drops (Cravit, Santan Inc. Japan) q.i.d., 1% prednisolone acetate eye drops (Predacetate, Allergan, India) q.i.d., and diclofenac sodium eye drops (Sinqi Inc., China) q.i.d for 1 week. At 1 day after surgery, BCVA became 1.4 (logMAR), while IOP was 18.0 mmHg. At 3 days after surgery, cornea edema was found, the IOP was increased to 50.0 mmHg, and further treatment was given, including intravenous infusion of 20% mannitol (250 mL) q.d for 3 days, oral acetazolamide tablets, eye drops with Brinzolamide (Azopt, Alcon Laboratories, Inc.) and carteolol hydrochloride (Otsuka Pharmaceutical Co., China) twice a day. At 1 week after surgery (Fig. 6), the IOP was decreased to 19.5 mmHg. Brinzolamide and carteolol hydrochloride were continued, pranoprofen eye drops (Pranopulin, Senju Inc., Japan) q.i.d were given, and oral acetazolamide tablets were stopped. During 1 to 3 months postoperative following-up, the IOP was maintained at 16.0–20.0 mmHg. The patient’s UCVA was improved from 2.0 to 1.0 (logMAR), and BCVA was improved from 2.0 to 0.5 (logMAR). Drugs for the left eye were discontinued.

No cataract surgery was given on the right eye for the personal reason except that the anti-glaucoma medication was continued to maintain a normal IOP.

## Discussion and conclusions

ARS represents a clinically and genetically heterogeneous group of developmental disorders, which primarily affect the anterior segment of the eye, often leading to secondary glaucoma. According to the traditional classification, ARS covers three subcategories: Axenfeld anomaly (e.g., posterior embryotoxon, and defect around anterior segment); Rieger anomaly (e.g., the iris changes like stromal hypoplasia and irregularly shaped pupils along with features mentioned in Axenfeld anomaly); and Reiger syndrome (e.g., Rieger anomaly and other systemic features). The diagnosis of this case should be Reiger syndrome since he presented ocular anomalies of Rieger anomaly together with systemic anomalies. His ocular abnormalities included as follows: small and flat cornea, corneal PMD, posterior corneal embryonic ring, iris atrophy, pupil displacement to the nose, abnormal anterior chamber angle development, cataract formation in his both eyes, and temporal iris hole formation of the left eye, while his systemic anomalies referred to midface abnormalities, hypodontia and redundant periumbilical skin. Although no obvious corneal embryonic ring was found under the slit lamp because of the extremely small cornea at first, we found the presence of corneal embryonic ring in the upper anterior chamber angle during the examination of gonioscopy. Furthermore, the patient had an anti-glaucoma surgery when he was 20 years old. We basically knew that the iris hole above his eyes was left during the surgery. However, it remained unknown whether this iris hole in the temporal side of his left eye was congenital or caused by the surgery? After careful observation by gonioscopy, the iris tissue around the temporal iris hole of the patient’s left eye was preliminarily determined to be congenital. After the communication with the chief surgeon of the anti-glaucoma surgery, it was further clarified that the hole was caused by congenital development. Besides, the iris matrix atrophy in both eyes was added to confirm the iris lesion.

The clinical manifestations of ARS are somehow similar to those of iridocorneal endothelial syndrome (ICE). ICE mainly includes corneal endothelium abnormalities and iris lesion, which is monocular mostly, and has no family history generally. Morphological and quantitative changes of corneal endothelium are often found through corneal endothelioscopy. This patient had binocular diseases with normal corneal endothelium, which was 2813/mm^2^, and such data were not consistent with the diagnosis of ICE. Another important disorder to consider is Peters anomaly, anirido goniodysgenesis, which is usually bilateral. Patients with this disease may also present posterior embryotoxon, iris coloboma, aniridia, small cornea, flat cornea and glaucoma as in ARS patients. However, the disease is characterized by the presence of one or more central corneal opacities caused by central absence of the corneal endothelium, Descemet’s membrane and posterior corneal stroma. The central corneal opacities are absent in ARS [9]. In this case, although the corneal diameter was only 7.8–8.8 mm and the corneal curvature was 38.2 D in the left eye, it was clearly defined as a small and flat cornea, while the central cornea was transparent.

A major consequence of the anterior segment dysgenesis is the increased IOP, leading to glaucoma in approximately half of the ARS patients [10]. Glaucoma onset mainly occurs before being a teenager. In Souzeau’s study, the median age at glaucoma diagnosis in 53 ARS patients is 13.5 years old, and its penetrance of glaucoma at age of 10 is 29.4% [11]. This patient was diagnosed with glaucoma and had an anti-glaucoma surgery due to visual field defect at the age of 20. Before the age of 20, the patient had no ocular symptoms and never used eye drops for long-term. The IOP of the patient was unstable before cataract surgery. The IOP was 25.0 mmHg at 9:00 am and 19.0 mmHg at 15:00 pm without IOP treatment. The IOP was 20.0 mmHg on the 1st day after cataract surgery, and it was increased to 50.0 mmHg on the 3rd day after surgery and then decreased to 19.5 mmHg after medication. At 1 month after surgery, the IOP of the left eye was stable with medication. During 1–3 months after surgery, the IOP was stable between 16.0–20.0 mmHg without medication. The increased IOP might be attributed to the abnormal development of the anterior chamber angle, which led to the obstruction of the outflow tract of aqueous humor, and the residual viscoelastic agent aggravated the outflow burden of aqueous humor.

ARS is genetically heterogeneous, and approximately 40% of ARS patients have been found to harbor mutations. The major candidate genes for ARS are PITX2 and FOXC1 [12]. FOXC1 belongs to the forkhead family of transcription factors, the biological function of which is to regulate migration and differentiation of mesenchymal cells in the embryogenesis [13]. In ARS, mutations in FOXC1 are related to ocular, maxillofacial and hearing defects [12]. PITX2 belongs to the bicoid-class of homeodomain protein family, and it is able to regulate the development of ocular anterior segment and several extraocular tissues, such as dentary [14]. In addition, it has also been reported that mutations in CYP1B1 [15] and PRDM5 [16] are associated with ARS. CYP1B1 belongs to cytochrome P450 super family of drug metabolizing enzymes and may play roles in the metabolism of substrates essential for ocular development [17]. PRDM5 protein belongs to PRDM protein family, and it is crucial for the development and maintenance of extracellular matrix (ECM), which may explain its involvement in the development of ARS [18]. In this case, a known pathogenic mutation NM_153427.2:c.272G > A was detected on the PITX2 gene; and an unknown mutation NM_001453.2:c.1063C > T was detected on FOXC1 gene. In PITX2 gene, NM_153427.2;c.272G > A; p.Arg91Gln;EX5E;Het: missense mutation has been reported in relevant literatures in patients [19, 20]. SIFT software was used to predict its effect on the protein function, and the result was defined as “damaging”. Furthermore, PITX2 gene was predicted to be pathogenic. PITX2 gene-related corneal circodermoma, iris dysplasia type 2, and ARS type 1 are all autosomal dominant inheritance, indicating that there is a harmful mutation on the allele that may lead to the occurrence of the disease. In FOXC1 gene, NM_001453.2;c.1063 C > T; p.Pro355Ser; EX1E;Het: missense mutation, no relevant literature has reported the pathogenicity of this site, and its clinical significance still remains unclear. In addition, FOXC1 gene was also predicted to be pathogenic by the algorithms of SIFT and PolyPhen software (categorized as ‘neutral’ and ‘deleterious’, respectively). This locus occurs very infrequently in populations. FOXC1-related iris dysplasia type 1 and ARS type 3 are autosomal dominant inheritance, indicating that there is a harmful mutation on the allele that may lead to the occurrence of the disease.

There are some limitations in the present study:

1. Only one ARS patient was reported because it was a type of rare autosomal dominant disorder in the ophthalmic clinic.
2. We did not know the entire family history of the patient and did not obtain the ocular and physical examinations from his parents, brother, sister, and his children. Moreover, we did not perform gene analysis for his family due to the cultural and economic reasons of the family members.

## Data Availability

All data generated and analyzed during this study are included in this article.

## Abbreviations

ARS: Axenfeld-Rieger Syndrome
BCVA: Best-corrected visual acuity
IOP: Intraocular pressure
PMD: Pellucid marginal degeneration
AXL: The axial length
APT: The average phacoemulsification time
MPE: The intraoperative measurements of phaco energy
ICE: Iridocorneal endothelial syndrome
ECM: Extracellular matrix
FOXC1: Forkhead box protein C1
PITX2: Paired-like homeodomain transcription factor 2
CYP1B1: CytochromeP4501B1
